# Indoxyl sulfate is associated with cognitive impairment in ESRD patients by activating the extrinsic apoptosis in the neuronal cells during differentiating process

**DOI:** 10.7150/ijms.109245

**Published:** 2025-03-10

**Authors:** Chih-Chuan Hsieh, Kuo-Cheng Lu, Chuen-Lin Huang, Jiun-Jie Wang, Ting-Yin Yeh, Shyh-Min Lin, Ya-Ling Chung, Yi-Chou Hou, Yuahn-Sieh Huang

**Affiliations:** 1 Department of Surgery, Zuoying Armed Forces General Hospital, Kaohsiung, Taiwan.; 2 Department of Neurological Surgery, Tri-Service General Hospital, National Defense Medical Center, Taipei City, Taiwan.; 3 Division of Nephrology, Department of Medicine, Taipei Tzu Chi Hospital, Buddhist Tzu Chi Medical Foundation, New Taipei City, Taiwan; Division of Nephrology, Department of Medicine, Fu-Jen Catholic University Hospital, School of Medicine, Fu-Jen Catholic University, New Taipei City, Taiwan.; 4 Department of Medical Research, Cardinal Tien Hospital, New Taipei City, Taiwan.; 5 Department of Physiology and Biophysics, National Defense Medical Center, Graduate Institute of Physiology, Taipei, Taiwan.; 6 Healthy Aging Research Center, Chang Gung University, Taoyuan, Taiwan.; 7 Department of Medical Imaging and Radiological Sciences, Chang Gung University, Taoyuan, Taiwan.; 8 Neuroscience Research Center, Chang Gung Memorial Hospital, Taoyuan, Taiwan Medical Imaging Research Center, Institute for Radiological Research, Chang Gung University.; 9 Graduate Institute of Life Sciences, National Defense Medical Center, Taipei City, Taiwan.; 10 Division of Radiology, Department of Medicine, Cardinal Tien Hospital, New Taipei City, Taiwan.; 11 Department of Medical Laboratory, Cardinal-Tien Hospital, New Taipei City, Taiwan.; 12 Division of Nephrology, Department of Internal Medicine, Cardinal Tien Hospital, School of Medicine, Fu Jen Catholic University, New Taipei City, New Taipei City, Taiwan.; 13 Department of Biology and Anatomy, National Defense Medical Center, Taipei City 11490, Taiwan.

**Keywords:** Cognitive impairment, End stage renal disease, indoxyl sulfate, extrinsic apoptosis, differentiating, choline acetyltransferase, memory loss, differentiation, Stem cell.

## Abstract

**Aim:** This study investigates the correlation between indoxyl sulfate (IS) levels and cognitive impairment in end-stage renal disease (ESRD) patients from human study, *in vivo* and *in vitro* study.

**Materials and Methods:** Comparison of demographic and biochemical data, including IS concentrations, was conducted between a control group(n=16) and the ESRD with cognitive impairment group (n=14) and without cognitive impairment (n=17). A CKD animal model induced renal impairment in adenine-fed C57BL/6 mice, assessing memory loss and behavioral changes. Immunohistochemistry evaluated choline acetyltransferase activity and GFAP expression. Differentiating SH-SY5Y cells were treated with IS, assessing cell viability and apoptosis via annexin V and propidium iodide staining and western blotting. Reactive oxidized species generation was measured using DCFCA fluorescence and NAC pretreatment.

**Results:** In ESRD patients with cognitive impairment, IS levels were significantly higher compared to healthy controls, along with older age. CKD mice exhibited renal impairment and memory loss, accompanied by altered choline acetyltransferase activity and GFAP expression. IS treatment induced early apoptosis in SH-SY5Y cells, associated with increased cleaved caspase 3 levels and Fas/Fas-ligand activity, altered Bax/Bcl2 ratio, and reactive oxidized species generation.

**Conclusion:** Elevated IS levels are associated with cognitive impairment and neuronal apoptosis, potentially mediated by oxidative stress. IS could be a therapeutic target for cognitive dysfunction in CKD, necessitating further research into its mechanisms and therapeutic interventions.

## Introduction

Cognitive impairment is known as the global health issue, which has aroused the huge socio-economic burden. The hallmark of the cognitive impairment is the chronic decline of memory. The chronic decline accompanies with the development of the comorbidities, including decreasing of activity in daily life or psychiatric disorders 1. Severe cognitive impairment may exacerbate the dementia or even the epilepsy. It has been known that several chronic illnesses are associated with the cognitive impairment, including the aging process, cerebrovascular disease, hypertension, diabetes mellitus or chronic kidney disease 1-3. It has been noticed that the pathogenesis of cognitive impairment might differ among different risk factors. For instance, the development of Alzheimer's disease is associated with the deposition of amyloid beta oligomers 4. The vascular dementia is associated with the post-stroke insult such as oxidative stress or neuron loss 5. The cerebrovascular attack and the microvascular damage could pose the neurodegenerative disorder in different time course. The microvascular damage is associated with the white matter damage along with the integrity of the blood brain barrier, and therefore the neurodegeneration could be exacerbated 6, 7. To restrain the chronical neuronal loss, the neuronal precursor cells could activate neurogenesis and migrate to the injured area The factors disturbing the repairing process might potentiate the severity of the neurodegenerative disorders 8.

Chronic kidney disease is defined as the chronic decline in glomerular filtration rate or the structural abnormality in urinary tract 9. Chronic kidney disease is an important risk factor for cognitive impairment/dementia. The incidence of cognitive impairment is higher in CKD patients than general population. The risk factors for cognitive impairment and CKD are in common, including the elderly, metabolic syndrome, hypertension, or other disorders. The complications of CKD, such as insulin resistance, hyperactivation of renin-angiotensin-aldosteronism, dysregulation of electrolyte, anemia, worsen the clinical manifestation of the cognitive decline in different manners 10, 11. Recently, the hazard by uremic toxin has been mentioned as the neurotoxic ability neurodegenerative disorders 11. Uremic toxins could be classified based on the molecular weight, the water solubility and the protein bounded characters 12. The clearance of protein bounded uremic toxin mainly depends on organic anion transporter on the proximal tubular cells and the glomerular filtration. In the patients with advanced CKD or end stage renal diseases, the clearance of protein bounded toxin is inferior with the therapy with hemodialysis or peritoneal dialysis 13. The neurologic hazards of protein bounded uremic toxin has been noticed recently. The protein bounded uremic toxin, such as indoxyl sulfate (IS), is associated with neuroinflammation by influencing the glial cell and disrupts the blood brain barrier vial the aryl hydrocarbon receptors. The most commonly postulated mechanism is the neuroinflammation by activating the astrocytes 14. However, the direct toxicity on the neuronal cell is scant.

Based on the previous studies, the incidence of cognitive impairment increased in the patients with chronic kidney disease/end stage renal disease 15. Our previous study also demonstrated axonal damage by axillary factors such as hypoalbuminemia, not by the generation of the traditional factors such as amyloid beta or tau deposition 16. The concentration of indoxyl sulfate was associated with the cognitive impairment in the patients with end stage renal disease. The aim of the study is to validate if indoxyl sulfate potentiates the neurodegenerative disorder vial disturbing the neuronal cell body.

## Materials and Methods

### Human study

#### Ethics and study population

The study was approved by the Institute of Research Board of Carinal Tien Hospital, New Taipei City, Taiwan (CTH-109-2-1-068) with the accordance of Declaration of Helsinki. The study had been performed from August 2019 to December 2020. The inclusion criteria for the study were: (1) the age over than 45 years old; (2) with verbal and written understanding of Chinses or Taiwanese. The exclusion criteria were listed as follows (1) age younger than 45 years; (2) recent stroke occurred within 6 months; and (3) aphasic or illiterate to Chinese or Taiwanese. After obtaining the the written informed consent from the participants, blood sampling and clinical assessment would be obtained. The participants were divided into 2 groups: control group and ESRD with cognitive impairment.

#### Cognitive assessment

In the study, we assessed the cognition with the Chinese version of the Mini-Mental State Examination (MMSE). A trained physician assessed the MMSE. for the participants with maintenance hemodialysis, we performed MMSE within the first hour of dialysis to avoid the hemodynamic variation. We defined cognitive impairment as MMSE scores among 10-24.

#### Measurement of indoxyl sulfate and amyloid beta 1/42 (Aβ 1/42)

The nonfasting venous blood sample were obtained from the participants. For the patients receiving maintenance hemodialysis, we obtained the blood sample on the midweek (Wednesday or Thursday). The measurement of indoxyl sulfate had been performed by with indoxyl sulfate ELISA kit (Leadgene Biomedical (Tainan city), US patent: US20150355171A1). The measurement of peripheral amyloid beta 1/42 was performed with immunomagnetic reduction (IMR) according to the references by Yang et al 17, 18. Briefly, the Immobilizing antibodies for reagent nanoparticles targeted Aβ 1/42 (Abcam/ab34376). Functionalized magnetic nanoparticles had a mean diameter of 50-60 nm. IMR reagents mixed with human plasma were analyzed using a superconducting quantum interference device (XacPro-S MagQu). Biomarker detection ranges of Aβ 1/42 was (1-30,000 pg/mL. Assay variability ranged from 7-10%, and mean values were used for statistical analysis.

#### Imaging Procedure

Images were acquired from 1.5T MR scanner (Trio, Magnetom, Siemens, Erlangen, Germany), including T1-weighted magnetization-prepared rapid acquisition gradient echo (T1-MPRAGE) and diffusion weighted images. In order to minimize the patient excessive motion, a fixation pad were used. The total acquisition time would be 45 minutes. Trio, Magnetom, Siemens. The imaging parameters for T1-weighted images were as follows: repetition time (TR)/echo time (TE) = 7242/3.052 ms, number of slices = 180, voxel size = 1 mm × 1 mm × 1 mm, inversion time = 900 ms, flip angle = 15°, matrix size = 224 × 256, field of view (FOV) = 224 mm × 256 mm.

Diffusion weighted images were acquired using a spin-echo echo planar imaging sequence with the following parameters: TR/TE/flip angle = 12000 msec/91.9 msec/90°, field of view = 19.2mm2, matrix size = 128 × 128. Diffusion-weighting gradients were applied in 64 noncollinear directions distributed over a full sphere and optimized using the static electron repulsion model. A b value of 1000 s/mm2 would be used in this study.

##### Image Analysis

Retrospective analysis of white matter hyperintensity is measured on diffusion imaging data with the model of Fixel-based analysis (FBA). Fixel-based analysis were carried out using single-tissue constrained spherical deconvolution in the freeware MRtrix3 according to its recommended protocol. The diffusion weighted images were denoised using Marchenko-Pastur principal component analysis. Gibbs ringing would be removed based on local subvoxel-shifts 19, and correction would be conducted for motion, distortion and bias field 20. The diffusion weighted images were resampled to an isotropic voxel size of 1.3 mm using cubic interpolation. Normalization will be performed to a group template by using non-linear co-registration. The white matter measures of (FD), Fiber Cross-section (FC), and the combination of both (Fiber Density and Cross-section, FDC) were calculated for each voxel.

### *In vivo* study

The protocol of animal study was approved by Institutional Animal Care and Use Committee from National Defense Medical Center, Taiwan (NDMC-IACUC-23-185). Eight-week-old male C57BL/6 mice were acquired from the BioLASCO Experimental Animal Center (Taiwan). The animals were housed five per cage with 50% ± 10% relative humidity at 24 ± 2°C and subjected to a 12-h light/dark cycle. The animals were acclimatized for 1 week prior to the start of experiments. The mice were fed a Purina chow diet with water ad libitum. The mice were randomly divided into the following six groups (6animals/group): chow-fed mice, adenine-fed diet (0.15%) for 4, 8 weeks and 12 weeks respectively.

#### Brain tissue IHC stain

The mice were anesthetized with isoflurane (2 %) and the animals were conducted cardiac perfused with 4 % paraformaldehyde. Tissues were incubated at 4 ℃ for post-fixation overnight. A brain was cut into 20-mm slices. The primary antibody against GFAP and choline acetyltransferase (CHAT) (Millipore Cat# AB144P) were applied to evaluate the staining. The mean intensity of immunohistochemistry was quantified in the region of interest (hippocampus and cortex) using ImageJ.

#### Novel object recognition test (NOR test)

The NOR test was performed to assess recognition memory and was performed during four consecutive days with a regular day/night cycle with standardization of time on each day. On the first two days of the protocol, mice were individually habituated to an empty arena (40 cm ×24 cm) during 10 min 21. On the third day (familiarization phase), two identical objects (brown-colored flasks) were placed 10 cm apart in the center of the arena and mice were allowed to freely explore the cage and objects for 5 min. On the fourth day (novel object phase) one object was replaced with a novel object (different color and shape, but similar in size). Mice were placed in the arena and again allowed to explore for 5 min. Trajectories and nose-point locations was recorded. Exploration time was defined as the time during which the nose-point is directed towards one of the objects with a proximity of 3 cm. The recognition index (time spent exploring novel object divided by total time exploring both objects) was calculated as a measure of recognition. Time spent investigating each object was scored using the behavior tracking software (Noldus EthoVision XT video tracking technology, version 17).

#### Transdermal glomerular filtration rate measurement

Glomerular filtration rate (GFR) was measured noninvasively by recording the transcutaneous fluorescence of FITC-sinistrin over time by attaching a fluorometer device (Medi-Beacon, St. Louis, MO, USA) to the mice. The fluorometer was affixed to the shaved back of anesthetized mice using a double-sided adhesive patch; the background fluorescence level of the skin was recorded for 1 minute, and FITC-sinistrin (15 mg/kg; Fresenius-Kabi, Linz, Austria) was then injected intravenously via the retro-orbital sinus. The fluorometer was programmed to make a transcutaneous measurement every 5 seconds; measurements were made for 5 hours and stored on the device. GFR was calculated using a single-compartment model, enabling direct conversion from the elimination half-life, using a published conversion factor.

#### 8-arm radial maze

The protocol for the eight-arm radial maze was executed according to previously established guidelines for the four-arm baited version 22. In summary, the maze apparatus comprised eight identical arms extending radially from an octagonal platform, elevated 80 cm above the floor and surrounded by external cues. Illumination was provided by overhead lighting, with 10 stimuli affixed to the curtain encircling the maze. Among these stimuli, five were shapes crafted from white cardboard (a star, rectangle, circle, and triangle), while the remaining five consisted of a yellow oval, a small red plastic butterfly, a small yellow plastic ball, and a black-and-white poster. Each arm was equipped with a cup containing food at its terminus. Habituation involved a single 10-minute exploratory trial to acclimatize the animals to the maze. Acquisition consisted of two consecutive 5-minute trials conducted daily over eight consecutive days. Retention trials, lasting 5 minutes each, were administered at 48-hour intervals following the completion of the previous session, spanning intervals of 48 and 96 hours. Arm entry was recorded when all four paws of the animal traversed the arm's threshold. Total errors were tallied as the sum of both reference and working memory errors. Reference errors were omitted from graphs displaying total errors. Differences among groups in latency to reach the four baited arms and in working, reference, and total memory errors were assessed in each trial.

### *In vitro* study

#### Cell line

The SH-SY5Y cells was purchased from the ATCC (ATCC® CRL-2266). Briefly, the cells were cultured every 3-5 days with confluence of 80% and then subcultured. The maintenance of the cell line was performed with Dulbecco's Modified Eagle Medium (DMEM):F12 1:1 medium with 8% fetal bovine serum (FBS, FBS, Tseng-Hsiang Life Science, Taiwan)), 2 mM L-glutamine, 100 IU/mL penicillin, 100 mg/mL streptomycin, 1 mM sodium pyruvate, and 1 mM nonessential amino acids at 37°C in a humidified atmosphere of 5% CO2. The indoxyl sulfate was purchased by Sigma Aldrich (Product number: I3875) with 5mM DMSO stock.

#### Differentiating agents and protocol

SH-SY5Y cell is the cell-line of the human neuroblastoma with differentiating ability to the mature neuron cells. To induce the differentiation, we used the differentiation protocol with retinoic acid (Catalog number: W519308, Sigma Aldrich) by Cheung et al 23. Briefly, cells were grown for 1 day prior to differentiation. The next day, 10 μM retinoic acid (RA) in DMEM with 3% FBS was applied; the medium with RA and 3% was changed every 3 days. After 7 days of differentiation the cells were used for the experiments. To mimicking the condition of the mature neurons and the neurons with differentiating status, we grouped the cells into three categories: (A) undifferentiated status: 8% FBS alone; (B) differentiated status: the cells treated with 3% FBS and 10 μM RA for 7 days; (C) differentiating status: the cells treated with 3% FBS and 10 μM RA for 1 days. Indoxyl sulfate had been treated for the undifferentiated SH-SY5Y cells for 24, 48 and 72 hours along with 8% FBS. Indoxyl sulfate had been treated for differentiated SH-SY5Y cells at the 8th day of 3% FBS with 10μM RA for 72 hours. The differentiating status is defined as the IS given on the 1st day of 3% FBS with 10μM RA in differentiating status for 24 and 48 hours respectively.

#### Cytotoxicity Assay

Cell viability is determined by the (3,4,5-dimethylthiazol-2-yl)-2,5-diphenylte-trazolium bromide assay (MTT, Catalog number: M6494, Invitrogen, United States). Briefly, 50 µL of the MTT labeling reagent (0.5 mg/mL as final concentration) was added to each well at the end of the incubation period and then placed in a humidified incubator at 37 °C with 5% CO2 and 95% air (v/v) for an additional 2 h period. The insoluble formazan was dissolved with DMSO, which converted the cells with yellow MTT tetrazolium into purple formazan product. The colorimetric determination of MTT reduction was determined at 540 nm. Control cells treated with DMEM-F12 were taken as 100% viability. We analyzed the cell viability with the following conditions: (a) the undifferentiated SH-SY5Y at 0 hours, 24 hours, 48 hours and 72 hours after been treated with IS at clinically relevant concentration; (b) the differentiated SH-SY5Y cells at 0 and 72 hours after been treated with IS at clinically relevant concentration; (c) the differentiating SH-SY5Y cells at 0 hours, 24 hours and 48 hours after been treated with IS at clinically relevant concentration.

#### Detection of reactive oxygen species

The intracellular reactive oxygen species was quantified by 2'-7'-Dichlorodihydrofluorescein diacetate (DCFCA, catalog number: E-BC-K138-F, Elabscience, Houston, Texas, United States) and flow cytometry. Briefly, SH-SY5Y cells (3 × 10^3^ cells per well) were cultured in 12-well tissue culture plates with 2′,7′-dichlorofluorescin diacetate, then the cells were cotreated with IS and 10μM RA for 1hours. SH-SY5Y cells were harvested and suspended in 1 × PBS buffer. Relative fluorescent intensities in differentiating SH-SY5Y were quantified using a flow cytometer (EPICS XL, Beckman Coulter, Fullerton, CA, USA). N-acetylcysteine (NAC) is regarded as the anti-oxidant agents. To measure the antioxidant effect, we pretreated the differentiating SH-SY5Y 30 minutes before the treatment of IS with NAC at 0.1mM and 0.3mM. MTT assay was performed after being treated after IS treated for 1 hour.

#### FITC Annexin V and Propidium Iodide apoptosis determniation

The flow cytometry analysis was performed by dual stain of Annexin V and PI (Cat. No.: 556547, BD bioscisences, San Diego, USA) to differentiate the mechanism cell death. After the IS treatment for differentiating SH-SY5Y cells, the cells were harvest by with cold DMEM. After removing the medium, we centrifuged the SH-SY5Y cells at 2500 rpm for 5 minutes at 4 degrees Celsius. We used PBS to wash the pellet and then stained the cells with Annexin V-FITC and PI in DMEM:F12 as the binding buffer. Five thousand events were collected on each sample. We used FACScalibur (Becton Dickinson, Mountain View, CA) in the FL1-H and FL2-H channels to analyze the stained cells.

#### Western blotting

Western blot analysis was performed as previously reported (Liu et al. 2014). Cells were washed twice with cold phosphate-buffered saline (PBS) after treatment, and total protein was prepared in radioimmunoprecipitation assay (RIPA) lysis buffer followed by centrifugation at 12,000 × g for 10 min at 4°C. Equal amounts of protein estimated using the BCA protein assay kit (Beyotime, Shanghai, China) were separated by SDS-polyacrylamide gel electrophoresis (SDS-PAGE) (8 to 12%) and transferred to nitrocellulose membranes (Millipore, Bedford, MA). The 5% milk was used for the blocking the blot at room temperature for 1 hours. Then the immune labeling was performed with primary antibodies as follows: cleaved caspase 3 (Cell signaling, Danvers, Massachusetts, United States; Catalog number: 9661, 1:1000 dilution); caspase 8 (Cell signaling, Danvers, Massachusetts, United States; Catalog number: 9746, 1:1000 dilution); Bcl-2 (Santa cruz, California, United States; Catalog number: 7382, 1:1000 dilution); Bax (Santa Cruz, California, United States; Catalog number: 7480, 1:1000 dilution); Fas (GeneTex, Zeeland, MI, USA, Catalog number: GTX31191, 1:500 dilution); Fas ligand (Genetex Zeeland, MI, USA, Catalog number: GTX31191, 1:500 dilution) and β-actin (Novus Biologicals, 1:1000 dilution). The membrane was incubated with diluted enzyme-linked antibody (1:1000 dilution) for 1 hour under the room temperature.

### Statistics

Continuous variables are presented as mean ± standard deviation. Categorical values are expressed as percentages. Student t test was performed due to compare the difference between 2 groups. Paired t test was applied to compare the difference of GFR, NOR test and 8-arm maze. A one-way analysis of variance was used to compare the differences in variables within the three patient groups. All statistical analyses were performed using the statistical package SPSS for Windows (v.17; SPSS, Chicago, IL, USA). A two-tailed p value of <0.05 was considered statistically significant.

## Results

### The correlation between the indoxyl sulfate and the cognitive impairment in ESRD patients with fiber decrease in stria terminalis

**Figure 1:**
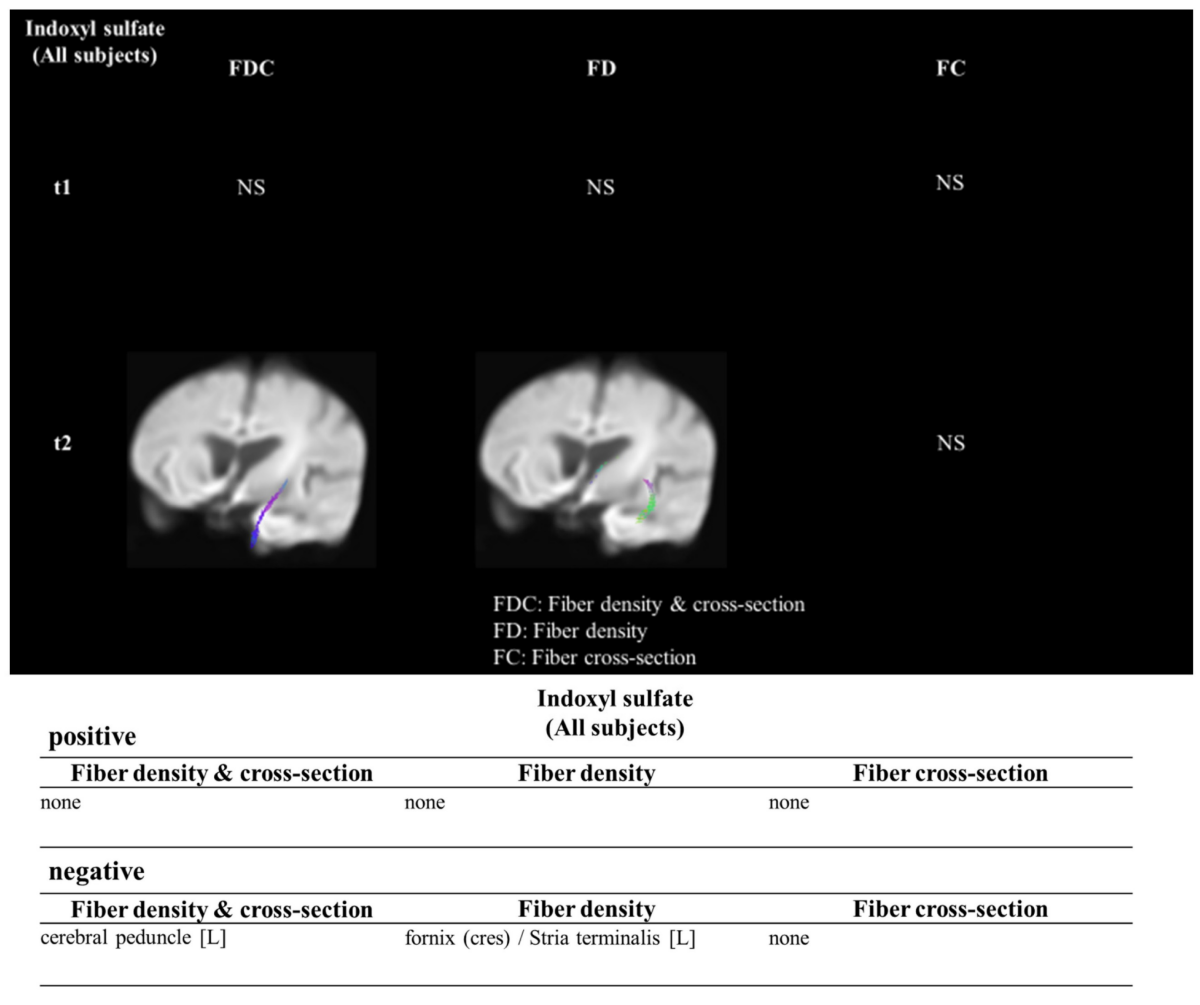
** The indoxyl sulfate and the influenced white matter density in the clinical study**. The Fixel based analysis demonstrated the association between the indoxyl sulfate and the decrease fiber cross section and density in the white matter.

Table 1 illustrated demographic results and the biochemical, hematologic result and the serum concentration of indoxyl sulfate between the control group(n=16) and the ESRD with cognitive impairment group (n=14) and without cognitive impairment (n=17). In the ESRD with cognitive impairment, the indoxyl sulfate concentration was 34.85 ± 15.36 μg/mL, which was higher than the control group (3.99 ± 7.60 μg/mL, p<0.001). The age was higher in the ESRD with cognitive impairment group in comparison with control group (74.80 ± 7.42 years old vs. 58.54 ± 8.79 years old, p=0.006). The ages between ESRD with and without cognitive impairment were similar (ESRD without cognitive impairment: 66.9 ± 7.2 years old, p=0.256). The Aβ 1/42 concentration was similar between groups (p=0.586). Figure 1 illustrated the association between the concentration of indoxyl sulfate and the fiber tracking study. The concentration of IS was negatively associate with the fiber density and fiber cross section in the strial terminalis.

### Memory loss developed in the CKD animal model along with the axonal damage

**Figure 2:**
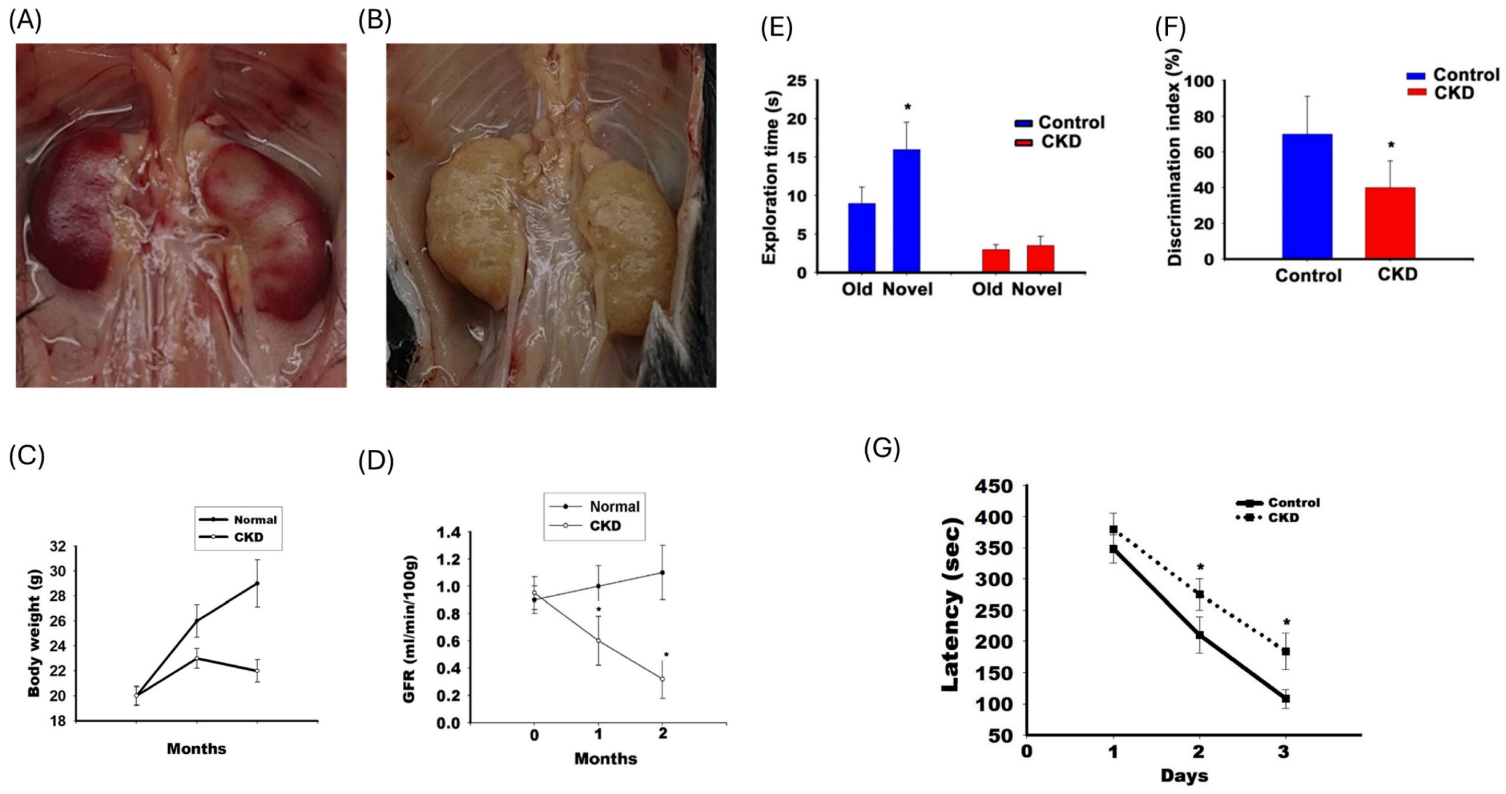
** The impairment in NOR test and 8-arm radial maze in adenine-fed mice. (A and B)** The gross character in and control mice and adenine-fed mice respectively. **(C)** The body weight change between the control and CKD mice. **(D)** illustrated the change in transdermal GFR between the control and CKD mice. **(E and F)** demonstrated the exploration time and discrimination index of the NOR test. **(G)** illustrated the latency between control and CKD mice in the 8-arm radial maze.

Axonal dysfunction is associated with the memory loss in the CKD animal model. Figure 2 illustrates the renal impairment in adenine-fed C57BL/6 mice. Panel A illustrates the gross characteristics of the kidney, which was decolorized and decreased in size in adenine-fed mice. Panel B illustrates the variation in TGFR for adenine-fed mice and control mice. Panel C illustrates renal function impairment in adenine-fed mice (labelled CKD). The GFR decreased from 0.92 ± 0.12 ml/min/100 g to 0.6 ± 0.17 ml/min/100 g at 1 month (compared with month 0, p <0.05) and 0.30 ± 0.15 ml/min/100 g at 2nd month (compared with month 0, p <0.05). This behavior was altered in a CKD animal model. Panels E and F show results of the NOR test. Panel E shows the exploration time for old and new objects in the control and CKD mice. The exploration time for old subjects and new subjects were 9.1 ± 2.3 and 16.1 ± 3.2 seconds respectively for control mice (with statistic difference, p<0.05). The exploration time for old subjects and new subjects were 2.3 ± 1.2 seconds and from to 3.5 ± 1.5seconds in CKD mice. Panel F shows the discrimination index in the CKD and control mice. The discrimination index was decreased in CKD mice (41 ± 17% in CKD and 71 ± 21% in control mice, p<0.05). Panel F shows the latency in the 8-arm radial maze. The latency time increased on 2^nd^ day (271.3 ± 12.6 seconds for CKD mice and 210.8 ± 20.3 seconds for control mice, p <0.05) and 3^rd^ day (201.3 ± 10.2 seconds for CKD mice and 110.4 ± 8.2 seconds for control mice, p <0.05).

**Figure 3:**
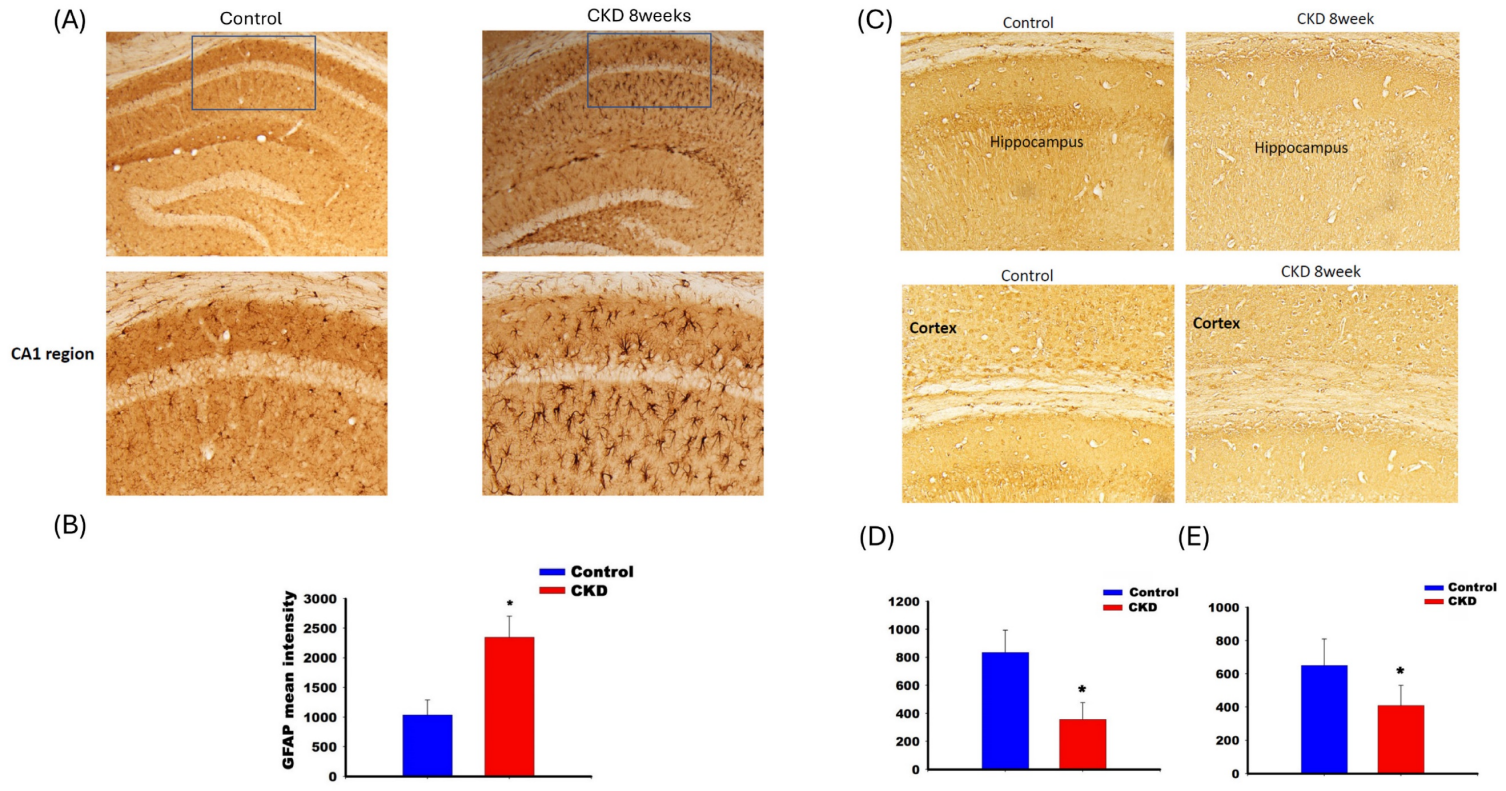
** The activation of GFAP of hippocampus CA1 region in adenine-fed mice at 8^th^ week (A).** The activity increased in the CA1 region of the CKD mice **(C)**. The decrease of choline acetyltransferase in CA1 of hippocampus **(D)** and cortex **(E)**.

Figure 3 shows the IHC staining for GFAP (panels A and B) and choline acetyltransferase (panels C, D, and E) in CKD mice. Panel A shows that GFAP expression increased in the CA1 region of the hippocampus. The expression of choline acetyltransferase decreased in the hippocampal CA1 region (panel D) and cortex (panel E) in adenine-fed mice for 8 weeks.

### The effect of indoxyl sulfate on the cell viability of SH-SY5Y cells during differenting process by triggering early apoptosis along with extrinsic apotosis

**Figure 4:**
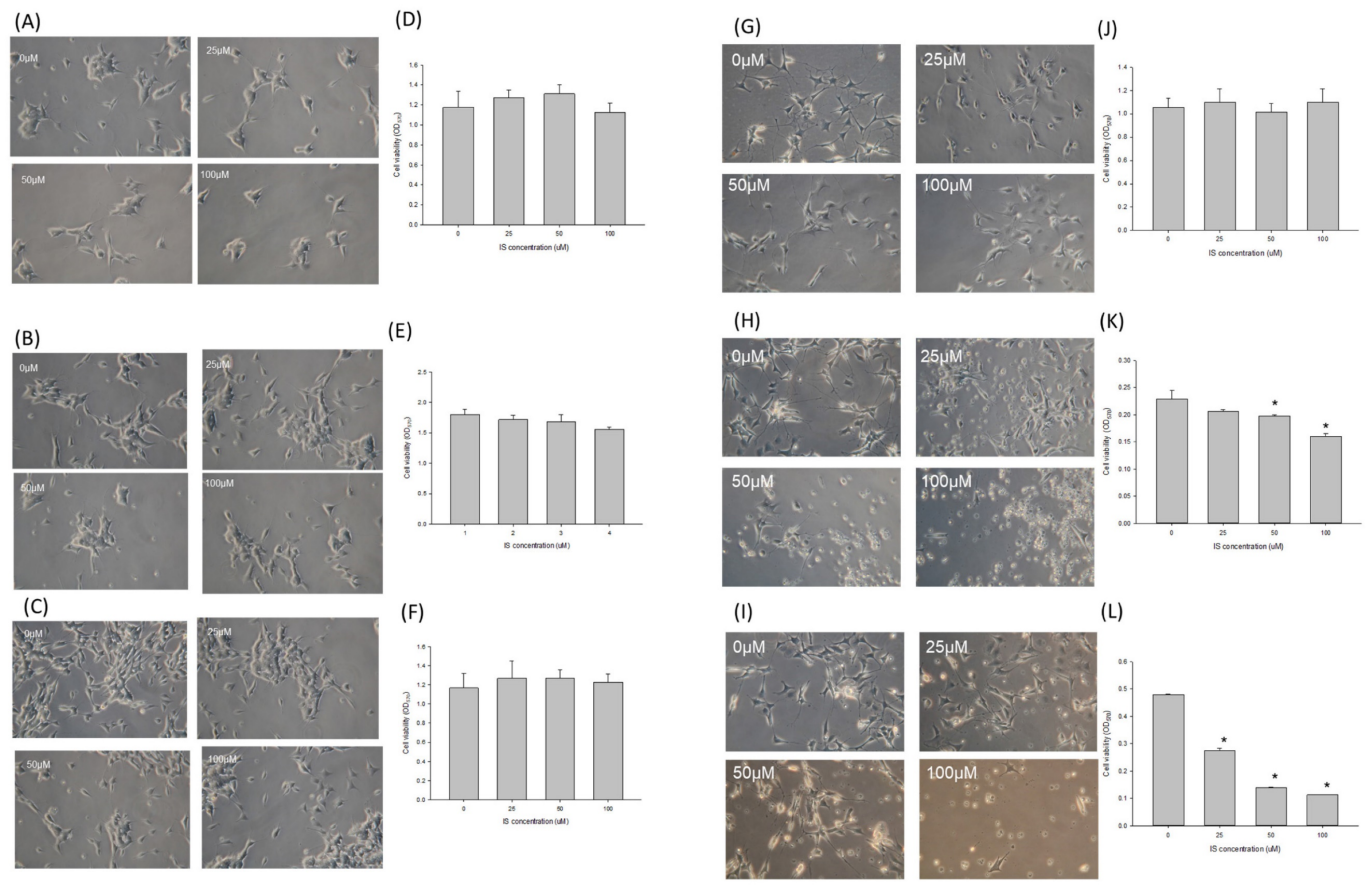
** The influence of IS on the cell viability in different differentiating status.** The effect of the indoxyl sulfate for the viability on undifferentiated SH-SY5Y cells after 24 **(A and D)**, 48 **(B and E)** and 72 hrs **(C and F)** respectively. The effect of indoxyl sulfate for viability on the differentiated **(G and J)** and differentiating SH-SY5Y cells at 24 hours **(H and K)** and 48 hours **(I and L)** respectively (*: p<0.05 in comparison with 0uM).

Figure 4 illustrated the morphology and cell viability of the SH-SY5Y after being treated with indoxyl sulfate under various differentiating status. Panel A to F demonstrated the cell viability of the undifferentiated SH-SY5Y when treated with IS at 0, 25, 50 and 100μM (panel A, B, C) for 24, 48 and 72 hours (panel D, E, F) respectively. In ESRD patients, impaired kidney excretion and inadequate dialyzer clearance may lead to increased indoxyl sulfate (IS) accumulation. Excess IS could oversaturate organic anion transporters (OAT), resulting in its accumulation in the CSF and potential brain damage. Potential mechanisms include dysfunctional OAT activity and oversaturation preventing IS clearance from the CSF 24, 25. Adesso et al. identified clinically relevant CSF IS concentrations of 15-60 μM, with plasma IS levels estimated to be 2.37 times higher 14. Based on this, we established a clinically relevant IS concentration of up to 100 μM. The cell viability was similar at 25, 50 and 100 μM when comparing with the control group at different time, which indicated that the IS didn't influence the cell viability for the undifferentiated SH-SY5Y cells. The panel G and J of figure 4 illustrated the morphology and cell viability by IS in differentiated SH-SY5Y cells. When IS was treated at the initial phase of differentiation, the cell viability decreased along in the dose-dependent manner of IS at 24 hours (panel H and K, p<0.05) and 48 hours (panel I and L, p<0.05).

**Figure 5:**
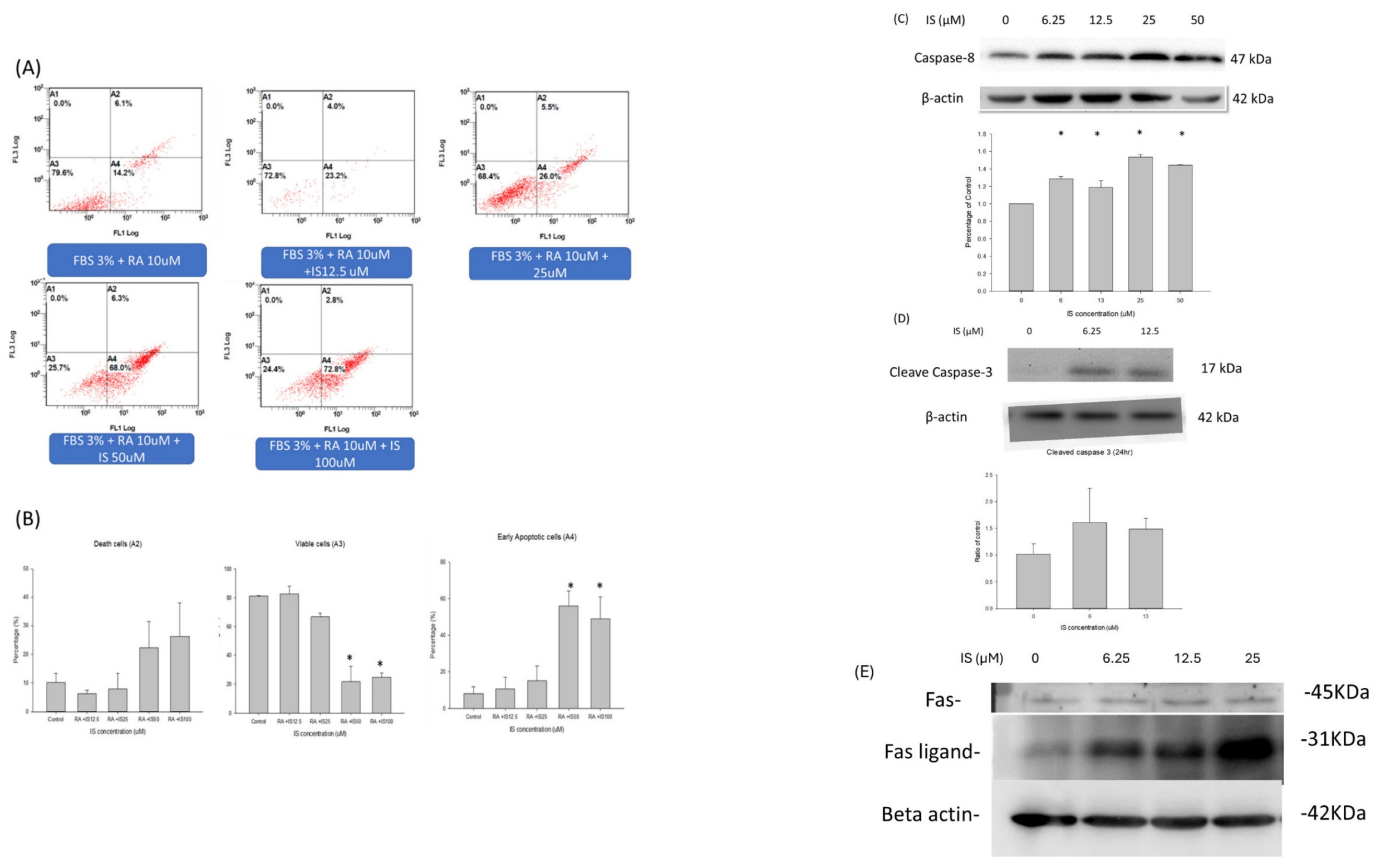
** The apoptosis profile in the flow cytometry and the caspase cascade. (A)** The annexin-PI dual stain of the differentiating SH-SY5Y cells treated with indoxyl sulfate. The flow cytometry of the dual stain for the SH-SY5Y cells treated with retinoic acid and IS at different concentration (0,12.5, 25, 50 and 100 μM). **(B)** The viable, death and early apoptotic cell counts between different concentration of IS. The activity of caspase 8 **(C)** and cleaved caspase 3 **(D)** in IS treated differentiating SH-SY5Y cells with different concentration of IS. Panel E demonstrated the western blotting of Fas/Fas-ligand for IS treated differentiating SH-SY5Y cells with concentration for 0,6.25, 12.5 and 25uM for 1hr.

Figure 5 illustrated the annexin V and propridium iodide stain for detection of apoptosis by flow cytometry for IS treated differentiating SH-SY5Y cells. Panel A illustrated the dual stain of differentiating SH-SY5Y cells with IS treatment at 0, 122.5, 25, 50 and 100 μM. The viable cells (A3) were decreased at the concentration of 25 μM, and the IS concentration at 50 and 100 μM decreased the percentage of the viable cells down to 20% in the total cell counts. In the early apoptotic cells (A4), the percentage increased as the concentration of indoxyl sulfate increased. The percentage of early apoptotic cells was up to 60% at the IS concentration 50 and 100 μM (p<0.05). The necrotic cells percentage(A2) also increased as the concentration of IS increased, but the difference was less statistically different. The data demonstrated that the IS treated differentiating SH-SY5Y cells had higher percentage of cell early apoptosis in a dose-dependent manner. Panel C and D displayed the western blotting for the caspase activity. The panel demonstrated the ratio of caspase 8 and beta-actin in the IS treated differentiating SH-SY5Y cells when treated at 3 hours at 6.25, 12.5, 25 and 50μM. The western blot for cleaved caspase 3 was demonstrated when differentiating SH-SY5Y cells were treated with IS for 6 hours. Only the concentration at 6.25 and 12.5μM were demonstrated because only the cell debris were seen in the SH-SY5Y cells treated at 25, 50 and 100μM. The cleaved caspase 3 increased at the 6.25 an 12.5 μM. The activity of caspase 9 was not detected in the western blotting. Panel E illustrated tha Fas/Fas-ligand activity of the IS treated differentiating SH-SY5Y cells for 1 hour. At 1 hours, the activity of Fas ligand increased with dose dependent manner from 0μM to 25μM.

### IS treated differentiating cells induced cell apoptosis vial alternation of mitochondrial Bax/Bcl2 homeostasis

**Figure 6:**
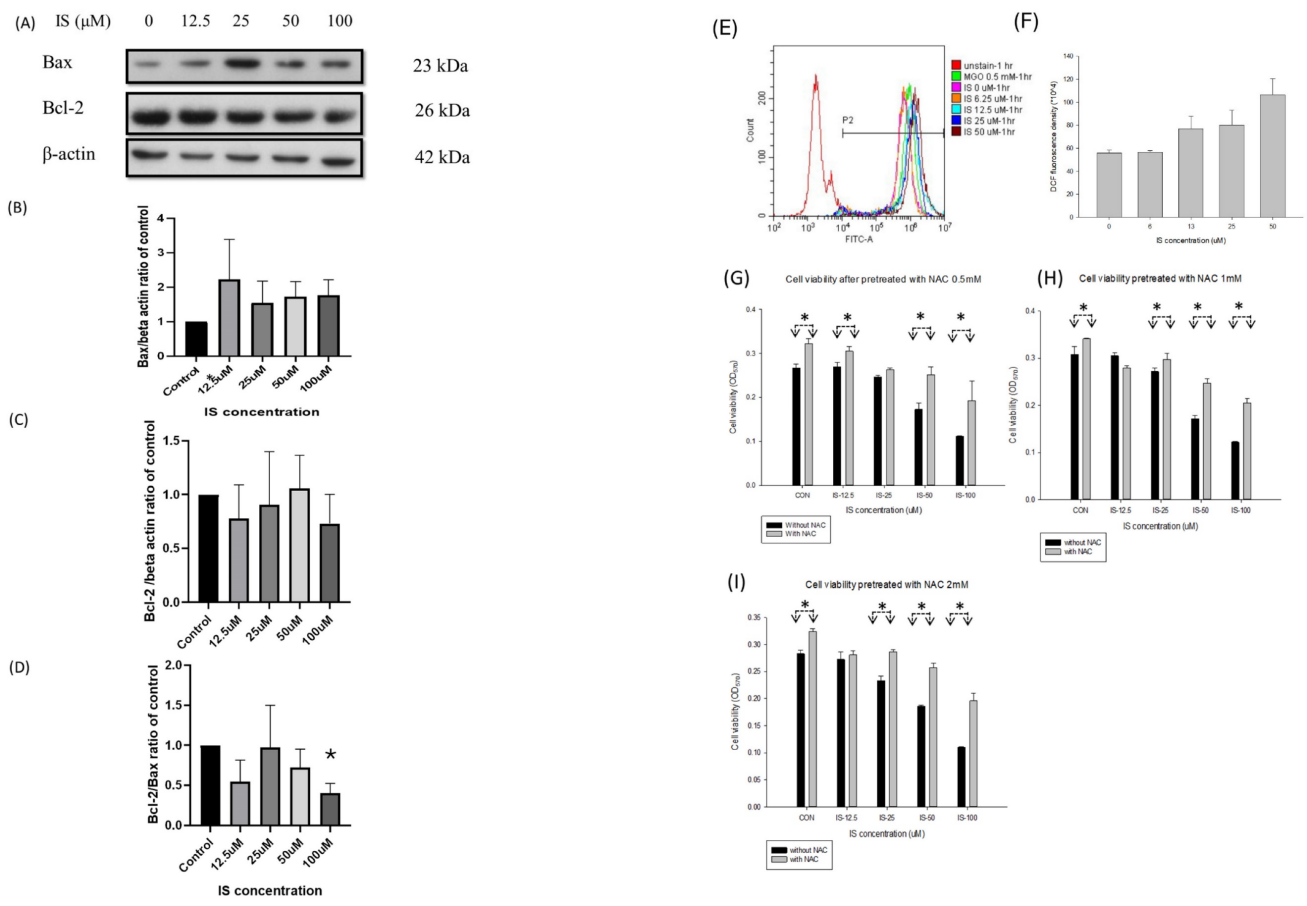
** The activity of Bax (B), Bcl-2 (C) and Bcl-2/Bax ratio (D) in IS treated differentiating SH-SY5Y cells with different concentration of IS.** The Bcl-2/Bax ratio decreased at 100μM (*: p<0.05, when compared with control group). **(E-I)** The production of DCFCA after being treated with indoxyl sulfate in differentiating SH-SY5Y cells with different concentration of IS. **(E)** The FITC at different concentration of IS along with the MgO. **(F)** The generation of DCF at different concentration of IS. **(G- I)** The cell viability after being treated with N-acetylcystein (NAC) at 0.5, 1,2 mM. The viability of the NAC-treated cell increased in comparison with the non-NAC treated cells at the same concentration (*: p<0.05, in comparison with the same concentration).

Figure 6 illustrated the activity of Bax and Bcl2 of IS treated SH-SY5Y cells for 6 hours. We used the western blotting to detect the expression of Bax, Bcl2 and Bcl-2/Bax ratio in the IS treated differentiating SH-SY5Y cells for 6 hours. From the IS concentration from 12.5 to 100 μM, the Bax expression increased in a dose dependent manner (panel B). The Bcl-2 expression decreased from at 12.5, 25 and 100μM, but increased at the concentration 50μM (panel C). The Bcl-2/Bax ratio decreased at the IS concentration 12.5, 50 and 100μM (panel D).

### The IS mediated apoptosis of differentiating SH-SY5Y cells is mediated by generation of reactive oxidized species

Panel E to I in Figure 6 illustrated the DCFCA of the IS treated differentiating SH-SY5Y cells. Methylglyoxal (MGO) at 0.5mM was served as the positive control for oxidative products. The DCFCA fluorescence density increased in a dose-dependent manner, and the density was higher at the concentration 50μM than control group (panel E and F). Panel G, H and I demonstrated that the cell viability of the IS treated differentiating SH-SY5Y cells if pretreated with 30-minute NAC at 0.5, 1 and 2 mM. The cell viability increased when comparing with the non-NAC treated cells at the IS concentration 12.5, 25,50 and 100μM at NAC concentration 0.5, 1 and 2 mM. In the pretreated concentration at 0.5, 1, and 2 mM, the cell viability increased at the IS concentration 100μM.

## Discussion

Based on our study, the concentration of indoxyl sulfate was associated with development of cognitive impairment in ESRD patients. From the FBA result, the concentration of IS was negatively associated with the fiber crossing the stria terminalis, where the neuronal progenitor cells reside for repairing the neuronal loss. In CKD animal model, the memory impairment developed along with the decrease in glomerular filtration rate by the 8-arm radial maze and the NOR test. From the *in vitro* study, the indoxyl sulfate decreased the SH-SY5Y viability only when initiating stage of the differentiation process. The decrease in viability was induced by the extrinsic apoptosis.

Based on the previous study, the plasma Aβ1/42 concentration was associated with the severity and the diagnosis of Alzheimer's disease 26, 27. In our study, the concentration was similar. The concentration of IS was higher in the ESRD with CI in comparison with the control group. For the IS concentration between the ESRD with and without cognitive impairment, a possible reason was the malnutrition status. It has been noticed that the malnutrition could worsen the cognition and as the risk factor of cognitive impairment 28, 29. Our study group also illustrated that the hypoalbuminemia was associated with the development of cognitive impairment 16. Indoxyl sulfate originated from the tryptophan, and the generation of IS might be lowered in the CKD patients with malnutrition or sarcopenia 30. Therefore, the IS concentration was lower in the ESRD with CI than without CI. But IS disturbed the blood brain barrier and then potentiate the neurodegenerative process by the malnutrition. Our radiologic finding demonstrated that the fiber connecting the stria terminalis decreased, and the indoxyl sulfate concentration was associated with the decrease in the fibers. Several studies illustrated the association between protein bounded uremic toxin, such as IS or p-cresol sulfate (PCS), was associated with cognitive impairment. To our understanding, this is the first result which correlates the protein bounded uremic toxin/indoxyl sulfate with the specific neurologic abnormality. Bed nucleus of stria terminalis (BNST) is the limbic forebrain structure with the connection with the anterior commissure 31. The caudal end of stria terminalis connects with stria terminalis, and the dorsal region connects with the septal and dorsal preoptic area. It has been known that the BNST function governs the neuroendocrine, autonomic and psychiatric function 32. Recent studies demonstrated that the BNST might influence the cognitive function vial the circuit-based mechanism. Lingg et al. demonstrated that the stimulation of the BNST input for the gray matter impaired the glucocorticoid dependent or independent consolidation of the memory in Sprague-Dawley rat 33. Beyond the autonomic and psychiatric function, it connects with the supraventricular zone which resides the progenitor cells. The neuronal progenitor cell plays an important role in repairing the injured neurons 34. It has been postulated that the blood brain barrier could be disrupted in the CKD by IS 25. On the other hand, the concentration of indoxyl sulfate in cerebrospinal fluid was associated with the neurologic complications in Parkinson disease or Parkinsonism 35. We postulated that the region with progenitor ability might be vulnerable for IS mediated toxicity.

Our animal study illustrated that the adenine-fed CKD model could activate the memory loss vial activating the GFAP activity and decreased the activity of the choline acetyltransferase activity. The activation of the GFAP reflected the activation of neuroinflammation 36. Previous *in vivo* study also demonstrated that the organic anion transporter on the blood brain barrier could actively transport the indoxyl sulfate out of cerebrospinal fluid to maintain the low concentration of IS 25. In CKD mice, the blood brain barrier was disturbed and therefore the increased influx of IS could induce further neurologic damage. Previous studies demonstrated the uremic toxin was associated with neurologic deficit in CKD animal by inducing the neuroinflammation within the astrocyte/glial cells. Adesso et al. illustrated that the IS aroused the neuroinflammation within the glial cells and the primary neuron cells vial activation of aryl hydrocarbon receptor and the Nuclear Factor-κB (NF-κB) 14. Sun et al. also illustrated that the PCS induced the neuroinflammation along with the behavior change 37. We firstly demonstrated the expression of neurotransmitter decreased in CKD animal model, and the behavior change, especially the legacy time in the 8-arm radial maze, increased in adenine-fed mice 38. The result reflected the spatial learning was impaired, which was consistent to the decrease in choline acetyltransferase activity in hippocampus CA1 region.

The *in vitro* study demonstrated that the IS potentiated the neurotoxicity for cells with differentiating ability. It has been known that the indoxyl sulfate was associated with other comorbidities, such as endothelial dysfunction or impairment cardiac remodeling. Such risk factors made the advanced CKD patients more vulnerable the ischemic stroke and sequential vascular dementia 39. Previous studies demonstrated that the neuronal progenitor cells migrate to the injured site and perform the neurogenesis vial endogenous recruitment process 7. Our *in vitro* model demonstrated the IS mediated neurotoxicity is mostly on the cells with neuronal differentiating ability. From the MTT assay, we demonstrated that the cell viability decreased only under the differentiating status of SH-SY5Y cells. The viability of both undifferentiated and differentiated status were similar when confronting the IS at 25, 50 or 100μM. The mature neuron is resistant to the stress from the indoxyl sulfate. A possible mechanism is that the glial cells are essential for neuroinflammation 40, 41). Our study design did not provide the interaction of the glial cells. Retinoic acid plays a critical role in driving the differentiation of SH‑SY5Y cells. In these cells, retinoid exposure not only triggers differentiation but induces oxidative stress during the differentiation process 42. This stress appears to activate FAS receptor-mediated pathways along with downstream effector caspases 3 and 8, processes that may be linked to the biogenesis or remodeling of essential organelles involved in apoptosis 43, 44. It has been known that the ROS regulates the cell signaling and the physiologic cell function 45. To initiate the differentiating process, oxidative stress is essential for stem cell self-renewal 46. The embryonic cells, with less mitochondria than mature cells, was under relatively hypoxic microenvironment. The increase of oxidative phosphorylation facilitated the ATP production and therefore shorten the G1 cell cycle and transition into the G2/M phase 47, 48. Besides, the sirtuin-1, as the inhibitor of p3-dependent function 49 is downregulated by endogenous ROS and therefore resulting in the further activation of developmental genes 50. Therefore, the DCFCA increase in SH-SY5Y cells treated with retinoic acid alone. From our model, the indoxyl sulfate activated the apoptosis with activation of caspase 3 and caspase 8 under the concentration of 6.25 to 12.5μM. The activation by caspase-8 cleaved BID and facilitated the release the truncated BID into the membrane of mitochondria, which thereafter altered the permeabilize the outer membrane of the mitochondria. The activated tBID/BAX complex then recruited the Bcl2 to the membrane and then result in inhibition of the apoptotic protein 51. The expression of Bcl-2 was highest at 25μM then decreased at higher concentration. The Fas/FasL expression increased in the IS treated cells at IS concentration higher than 12.5uM. From the finding, the extrinsic apoptosis pathway was initiated in the apoptosis of differentiating SH-SY5Y cells at IS concentration more than 25μM. Based on the result of DCFCA and the formation of Bcl2/ Bax, the intrinsic apoptosis could be observed in IS treated SH-SY5Y cells 52. The extrinsic apoptosis is usually liganded by the death receptor and the downstream formation of death signal complex 53. A possible mechanism is that the Fas/FASL activation could be mediated by the activation of aryl hydrocarbon receptor in specific cells 54. The expression of Fas/FASL was also observed in the pontosubicular necrosis in the neonatal neurons 55. Moreover, the caspase-dependent signal is essential for cellular differentiation, as the FAS/caspase cascade not only initiates apoptotic signaling but also contributes to organelle remodeling during differentiation 56. Additionally, investigations in SH‑SY5Y cells have shown that N‑acetylcysteine (NAC) can inhibit the activation of caspases 3 and 8, suggesting its potential to modulate the apoptotic cascade induced during differentiation 57. Based on the result, we postulated the IS might diffuse into brain parenchyma in a more facilitated manner and therefore decease viability of neuronal cells with repairing ability. Basic study demonstrated the efficacy of dialysis on the clearance on the Aβ 58. But the renal replacement therapy might not facilitate the excretion of indoxyl sulfate and therefore delay the neurodegenerative process. Therefore, the therapy focusing on the stem cell or regeneration neuron might provide the preventive strategy for mitigating the cognitive decline in ESRD/adverse CKD patients.

### Study limitations

Several limitations lie in our study. First, we compared the concentration of IS between the health control and ESRD patients with cognitive impairment in this case-control study. To further validate the effect of IS by the dose-dependent manner and the causal temporality, the patients with different stage of CKD and larger sample size might be needed. Besides, we determine the cognitive impairment based on the MMSE scale. Although the Aβ 1/42 concentration, as the biomarkers of Alzheimer's disease, were similar between groups, other diagnostic tools should be included to distinguish different types of the neurodegenerative disorders. Second, the *in vivo* study for linking clinical evidence and the *in vitro* evidence of the IS mediated neurotoxicity during differentiation is not performed. The animal model in CKD (eg. the 5/6 nephrectomized mice or adenine -fed mice) or the AhR knockout mice might be helpful in understanding the IS mediated toxicity in different stage of neuron. Third, we focused on the neurotoxicity with the apoptosis in the in differentiating SH-SY5Y cells. However, the influence of IS on neurologic function, such as the AchE activity or the release of neurotransmitter, were not measured. Further studies might be needed.

## Conclusion

The serum concentration of indoxyl sulfate was associated with the clinical cognitive impairment in patients with end stage renal disease. The activity of choline acetyltransferase decreased along with the spatial memory in CKD animal model. The IS decreased the viability of differentiating SH-SY5Y cells. The enhanced oxidative stress by IS activated the extrinsic apoptosis pathway for the differentiating SH-SY5Y cells.

## Figures and Tables

**Table 1:** The demographic data, MMSE and laboratory data of the participants

	Control	Dialysis		
		Normal Cognition	Abnormal Cognition	P value
Age (years)^b^	61.1±10.4	66.9 ± 7.2	72.4 ± 9.4	0.006*
Sex (M/F)	11/5	10/7	7/7	0.57
Education (years)^a,b^	13.0 ± 1.8	9.9 ± 3.9	8.3 ± 4.6	<0.001*
Hypertension (-/+)	8/8	3/14	2/12	0.05
Diabetes (-/+)	10/6	6/11	8/6	0.25
MMSE^b,c^	29.9 ± 0.3	28.6 ± 2	20.4 ± 4.4	<0.05
Amyloid beta (Aβ 1/42) (pg/mL)	16.42 ± 0.44	16.45 ± 0.41	16.56 ± 0.44	0.586
Indoxyl sulfate(ug/mL)^ab^	3.99 ± 7.60	43.04 ± 13.90	34.85 ± 15.36	<0.001*

*: p<0.05 a: Control versus Normal cognition in Dialysis; b, Control versus abnormal cognition in Dialysis; c, Normal cognition in Dialysis versus abnormal cognition in Dialysis

## Abbreviations

Aβ 1/42: Amyloid beta 1/42
BSNT: Bed nucleus of stria terminalis
CKD: Chronic kidney disease
DCFCA: 2'-7'-Dichlorodihydrofluorescein diacetate
GFR: estimated glomerular filtration rate
ESRD: end stage renal disease
GFAP: glia fibrillary acid protein
IS: indoxyl sulfate
MGO: Methylglyoxal
MMSE: Mini-Mental State Examination
MTT: (3,4,5-dimethylthiazol-2-yl)-2,5-diphenylte-trazolium bromide assay
NAC: N-acetylcysteine
NOR test: Novel object recognition test
RA: retinoic acid
